# Biotipo facial en relación a la forma del arco dentario superior de individuos de la ciudad de corrientes. un estudio transversal

**DOI:** 10.21142/2523-2754-1102-2023-151

**Published:** 2023-06-29

**Authors:** María C. Affur, Gabriela G Bessone

**Affiliations:** 1 . Facultad de Odontología, Universidad Nacional del Nordeste. Corrientes, Argentina. mcaffur@odn.unne.edu.ar, gbessone@odn.unne.edu.ar Universidad Nacional del Nordeste Facultad de Odontología Universidad Nacional del Nordeste Corrientes Argentina mcaffur@odn.unne.edu.ar gbessone@odn.unne.edu.ar

**Keywords:** cara, arco dental, índice, población, face, dental arch, index, population

## Abstract

Este trabajo de investigación buscó definir un patrón antropométrico facial que caracterice a los individuos de la ciudad de Corrientes.

**Objetivos:**

Obtener la morfometría facial de individuos de ambos sexos y determinar su frecuencia. Determinar los tipos de arcos maxilares más frecuentes en la muestra. Analizar la variación facial a través del índice morfométrico con relación a la forma del arco maxilar.

**Materiales y métodos:**

El estudio fue observacional, descriptivo y transversal. Se analizó la variabilidad morfométrica facial en relación a la forma del arco dentario superior, que presentó un grupo de 50 pacientes de ambos sexos, nacidos en la ciudad de Corrientes, Argentina, con un rango de edades entre 18 a 40 años, que concurrieron voluntariamente para su atención al Hospital Odontológico Universitario de la FOUNNE y al SAPS Rossi Candia, dependiente de la Municipalidad de la Ciudad de Corrientes. Para establecer el Índice Morfológico Facial (IMF), se implementó la medición de Rakosi e Irmtrud, utilizando un calibrador digital con un rango de 150 mm. Para determinar la forma de los arcos, se elaboraron modelos de yeso obtenidos de cada maxilar perteneciente a la muestra y con la utilización de plantillas marca 3M se clasificaron los arcos, según Williams. Con la forma del arco superior ya establecida se hicieron estudios de correlación con los biotipos faciales. Se trabajó a un p < 0,05.

**Resultados:**

El biotipo facial más frecuente fue mesoprosopo, con 38%; seguido por leptoprosopo, con 36%, y euriprosopo, con 26%, Respecto de la forma del arco, el 46% de los pacientes presentó forma de arco cuadrangular; el 40%, ovoide, y el 14%, triangular. Ninguna de las variables estudiadas se correlacionó significativamente con la edad de los pacientes. Los valores calculados para chi cuadrado no resultaron significativos (p-valores > 0,05).

**Conclusiones:**

Los resultados obtenidos no permiten establecer un patrón antropométrico facial específico en los individuos de la ciudad de Corrientes. Asimismo, existe variabilidad morfométrica facial con respecto a la forma del arco dentario superior.

## Abstract (en)

This research sought to define a facial anthropometric pattern that characterizes individuals from the City of Corrientes.

**Objectives:**

Obtain the facial morphometry of individuals of both sexes and determine its prevalence. To determine the most frequent types of maxillary arches in the sample. To analyze the facial variation through the morphometric index in relation to the shape of the maxillary arch.

**Materials and methods:**

This study was observational, descriptive, and cross-sectional. The facial morphometric modification was analyzed in relation to the shape of the upper dental arch, which was presented by a group of 50 patients of both sexes, born in the city of Corrientes, Argentina with an age range between 18 to 40 years, who voluntarily attended for his attention to the FOUNNE University Dental Hospital and the Rossi Candia SAPS dependent on the Municipality of the City of Corrientes. To establish the Facial Morphological Index (FMI), the Rakosi & Irmtrud measurement was implemented, using a digital caliper with a range of 150 mm. To determine the shape of the arches, plaster models obtained from each maxilla belonging to the sample are made and with the use of 3M brand templates, the arches are classified according to Williams. With the upper arch shape, correlations with facial biotypes have already been established. (p<0.05).

**Results:**

The most frequent facial biotype was Mesoprosopo with 38% followed by Leptoprosopo 36% and Euryprosopo 26%. Regarding the arch shape, 46% of the patients studied presented a quadrangular arch shape, 40% Ovoid, and 14% Triangular. None of the variables studied was significantly correlated with the age of the patients. The values calculated for Chi-Square were not significant (p-values > 0.05).

**Conclusions:**

The results obtained do not allow for establishing a specific facial anthropometric pattern in individuals from the City of Corrientes. Likewise, there is facial morphometrics regarding the shape of the upper dental arch.

## INTRODUCCIÓN

La antropometría es la ciencia que estudia las medidas del hombre. Se refiere al estudio de las dimensiones y proporciones de las partes del cuerpo humano, con el propósito de comprender los cambios físicos del hombre y las diferencias entre sus razas. Se originó durante el renacimiento y definió sus métodos por las reglas de expresión del neoclasicismo ^1^. A su vez, la antropometría facial estudia específicamente las medidas de la cabeza y la cara. La antropometría experimentó un renacimiento hace tres décadas, cuando los médicos comenzaron a usar las medidas de la superficie de pacientes con deformidades craneofaciales congénitas o adquiridas ^2^.

El examen clínico extraoral determina el tipo facial y el examen clínico intraoral, la forma del arco dental, dos características importantes para tener en cuenta en el diagnóstico integral del paciente. La relación entre las características faciales y la forma de los arcos dentarios ha sido analizada por diferentes autores, determinando que los arcos dentales de pacientes dolicofaciales son angostos y pueden estar asociados con una bóveda palatina alta ^3^; no obstante, hay poca correlación entre los anchos de arco y cualquier medición del ancho esquelético facial ^4^, datos históricos importantes que resultan relevantes para continuar investigando está relación.

En este sentido, la relación entre las dimensiones del arco dental y las estructuras craneofaciales en adolescentes con maloclusión clase II división 1 demostraron que los dolicofaciales tienen un arco cónico y los braquifaciales, un arco ancho con predominio en sujetos masculinos ^5^. Las formas y tamaños del arco mandibular; en cambio, tienen características matemáticas similares, en los tres tipos faciales, pero no se observaron diferencias significativas entre niñas y niños, en la población estudiada.

Otros estudios determinaron la correlación entre el índice facial, el índice cefálico, la longitud y el ancho del arco dental maxilar y mandibular. Los autores encontraron una mayor prevalencia de caras según el índice facial morfológico: un 35,6% fueron leptoprosopos y un 24,5%, hiperleptoprosos; mientras que, para el índice craneal, un 35,8% fueron mesocéfalicos y un 30,2%, braquicéfalicos; y en la forma trapezoidal de arco maxilar y mandibular fue del 60,4%. El índice facial mostró correlación con las medidas lineales del largo maxilar y mandibular ^6^. Asimismo, un estudio de la relación existente entre el tipo facial vertical y el arco dental ha determinado que los individuos de cara larga presentan arcos dentales estrechos y los de cara corta presentan arcos dentales anchos ^7^.

Las medidas faciales han sido variables fundamentales para determinar las características específicas de ciertas razas, ^8^^-^^16^ establecer reglas para dibujar el rostro y la cabeza, y, de manera más reciente, se han desarrollado aplicaciones en interfaces hombre-máquina a través del reconocimiento automático de rostros. 

Las investigaciones realizadas sobre el tema no muestran datos que revelen las características morfológicas faciales de los individuos de la ciudad de Corrientes y tampoco se han encontrado antecedentes relacionados a la prevalencia de forma y tamaño de los maxilares, en esta población. Por tal motivo, el propósito de esta investigación fue establecer un patrón morfológico facial y la forma maxilar más frecuente que caracterice a los individuos de la ciudad de Corrientes, con el fin de generar nuevos conocimientos y proporcionar datos relevantes que contribuyan con las ciencias biológicas, antropológicas y forenses. 

## MATERIALES Y MÉTODOS

Este estudio fue de tipo observacional, descriptivo y transversal, éticamente fue aprobado por el Comité de Bioética de la Investigación de la Facultad de Odontología de la Universidad Nacional del Noreste UNNE por dictamen N° 144/2020 y avalado por el Consejo Directivo de la FOUNNE, según Resolución N° 144/20 CD. Se trabajó con 50 pacientes que concurrieron a la Sala de Atención Primaria de la Salud Dr. M. Rossi Candia de la ciudad de Corrientes y al Hospital Odontológico Universitario de la Facultad de Odontología de la Universidad Nacional del Nordeste, previa autorización de los respectivos directores y coordinadores de los servicios, durante los años 2018-2019, de ambos sexos, nacidos en la ciudad de Corrientes cuyo rango de edades osciló entre 18 y 40 años.

Los pacientes fueron seleccionados teniendo en cuenta los siguientes criterios de inclusión, piezas dentarias permanentes en boca hasta el primer molar que no hayan recibido tratamiento de ortodoncia y ortopedia previo al estudio. Se excluyeron pacientes con alteraciones dentarias, de crecimiento y desarrollo que afectaran el sistema estomatognático y pacientes con asimetrías faciales en el plano transversal.

Los datos obtenidos de cada paciente fueron volcados de manera secuencial en una hoja de datos que constaba con la siguiente información, número de registro, edad, sexo, medida Nation-Gnacion (NGN), medida cigomático derecho a cigomático izquierdo (CGDI), longitud total del arco (LONARC), longitud anterior del arco (LONANT), ancho anterior del arco (ANCHCC) y ancho posterior del arco (ANCHMM). 

Para obtener el Índice Morfológico Facial (IMF) se realizaron mediciones directas, con un calibrador digital electrónico, marca Miyoshi, con un rango de 0-150 mm. Para ello el paciente se encontraba sentado en la unidad odontológica, con la cabeza erguida y en esa posición se realizaron los registros, aplicando la medición de Rakosi e Irmtrud que consistió en obtener dos medidas clínicas de la cara incluyendo: 1) del Nasion (N) a Gnation (GN) y 2) de Cigomático derecho (Cgd) a Cigomático izquierdo (Cgi). 

El IMF, se calculó a partir de la relación entre la altura morfológica de la cara (NGN) y la anchura bicigomática (CGDI), aplicando la siguiente fórmula: 


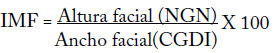


El IMF permitió determinar el tipo facial existente en la muestra estudiada los que fueron. Euriprosopo (EUR): son más dominantes las dimensiones transversales que las verticales, y eso da a la cara un aspecto más cuadrado y robusto, IMF entre 80,0-84,9. Leptoprosopo (LEP): cuando exhibe una forma facial estrecha y larga, con una mayor predominancia de las dimensiones verticales y un IMF entre 90,0-94,9. Mesoprosopo (MES): se caracteriza por tener una anchura y altura facial equilibradas y cuyo IMF está entre 85,0-89,9 ^8^.

Luego, a cada paciente se le realizó la toma de una impresión del maxilar superior con alginato, utilizando cubetas de stock apropiadas, las que posteriormente fueron vaciadas con yeso densita. Sobre los modelos obtenidos, se realizaron con regla Viner las siguientes mediciones, distancia intercanina (ancho anterior) y la distancia intermolar (ancho posterior). Una vez obtenida dichas medidas se dividió el valor de la distancia intercanina por el valor de la distancia intermolar y el resultado se expresó en porcentaje. Finalmente, para establecer la forma según la clasificación descrita por Williams, se calculó la media aritmética y la desviación estándar, de tal manera que todos los valores iguales a la media y que estén dentro del rango de desviación estándar correspondían a la forma de arco ovoidal, los valores mayores correspondían a la forma cuadrangular y los menores a la triangular. Por otra parte, se utilizaron plantillas marca 3M, con las tres formas seleccionadas y mediante observación directa se registraron los datos obtenidos, con el propósito de relacionar los valores con la forma que pueden adoptar los arcos. 

Todos los registros obtenidos fueron relacionados con sexo y edad de los individuos que conformaron la muestra. Las mediciones se registraron en planilla Microsoft Excel 2010 para luego analizarlas mediante la utilización el *software* InfoStat 2018 ^16^. Las pruebas de asociación fueron trabajadas con el estadístico de chi cuadrado p < 0,05.

## RESULTADOS

En el estudio se incluyeron 50 pacientes, de los cuales, el 58% fueron mujeres ^29^ y un 42% de varones ^21^. Ambos grupos presentaron una distribución similar en cuanto a las edades, las que se encontraron en un rango de 19 a 27 años. Las pruebas estadísticas aplicadas no mostraron valores significativos en relación a la variante de edad. (p < 0,05).

Los datos obtenidos se analizaron mediante el estadístico chi cuadrado para las variables biotipo facial y forma de arcada en relación con el sexo y entre ellas, para establecer su distribución. Los valores de chi cuadrado y sus correspondientes p-valores se presentan en la Tabla 1.

**Tabla 1:** Valores del estadístico chi cuadrado y p-valores asociados, para la prueba de Independencia entre las variables biotipo facial y forma de arco respecto del sexo y entre ellas Variable 1

Variable 1	Variable 2	p-valor
Biotipo facial	Sexo	0,921
Forma de arco	Sexo	0,600
Biotipo facial	Forma de arco	0,162

Los valores de chi cuadrado calculados no resultaron significativos (p-valor > 0,05), por lo que se puede advertir que no existe asociación entre los valores de biotipo facial y forma de arco y el sexo.

En cuanto al biotipo facial, según el sexo del paciente, las tablas de contingencia con las frecuencias encontradas para las diferentes combinaciones arrojaron los siguientes valores (Tabla 2).

**Tabla 2:** Tabla de contingencia entre las variables biotipo facial y sexo

Sexo	Euriprosopo		Leptoprosopo		Mesoprosopo		Total
Femenino	7	24%	11	38%	11	38%	29
Masculino	6	29%	7	33%	8	38%	21
Total	13		18		19		50

En el sexo femenino se encontró un 24% de euriprosopos, un 38% de leptosprosopos y un 38% de mesoprosopos. En los hombres se presentó un 29% de euriprosopos, un 33% de leptosprosopos y un 38% de mesoprosopos. No obstante, estas pequeñas diferencias no resultaron significativas, de acuerdo con lo establecido en la prueba de chi cuadrado (p > 0,05).

Se aplicó la misma prueba relacionando las variables FORMA y sexo hallando los siguientes valores (Tabla 3).

**Tabla 3:** Tabla de contingencia entre las variables forma de arco dentario superior y sexo

Sexo	Cuadrangular		Ovoide		Triangular		Total
Femenino	15	52%	10	34%	4	14%	29
Masculino	8	38%	10	48%	3	14%	21
Total	23		20		7		50

Asimismo, en relación con el sexo femenino, un 52% presentó forma cuadrangular; un 34%, arcos ovoidales, y un 14%, triangulares. En el sexo masculino, se encontró un 38% de formas de arco cuadrangulares, un 48% de ovoidales y un 14% de triangulares. No obstante, estas pequeñas diferencias no resultaron significativas, de acuerdo con lo establecido en la prueba de chi cuadrado (p > 0,05).

Pese a que no se han encontrados diferencias significativas en cuanto a las variables biotipo facial y forma con el sexo del paciente, se aplicó un análisis de la varianza y posterior prueba de F a fin de comparar los promedios de las variables cuantitativas respecto del biotipo facial y forma de arco dentario (Tabla 4).

**Table:** 

Variable	Biotipo facial		Forma de arco	
F	p-valor	F	p-valor
Altura facial	7,96	0,001 *	1,38	0,261
Ancho facial	2,29	0,112	0,31	0,731
Índice morfológico facial	134,67	<0,001 *	1,21	0,308
Longitud de arco	0,07	0,928	2,10	0,133
Ancho de arco	2,74	0,074	0,91	0,410
Ancho medio de arco	1,31	0,280	1,24	0,299
Longitud anterior de arco	1,06	0,354	59,46	<0,001 *
Índice morfológico facial 1	1,42	0,251	2,93	0,063
Índice morfológico facial 2	0,70	0,503	1,43	0,250

Valores seguidos de (*) que resultaron estadísticamente significativos.

Al analizar los valores del estadístico F y el p-valor para el biotipo facial resultaron estadísticamente significativos los valores de la altura facial y del índice morfológico facial, mientras que, para la forma del arco, solamente resultaron significativas las medidas de longitud anterior del arco. 

Al relacionar la forma del arco superior con los biotipos faciales se pudo establecer que la forma triangular se corresponde al biotipo facial euriprosopo, la ovoidal con el biotipo facial leptoprosopo y la forma cuadrangular se vinculó directamente con el mesoprosopo. Sin embargo, el biotipo facial no alcanzó asociación significativa con la forma del arco superior (p > 0,05).

## DISCUSIÓN

Las mediciones y análisis realizados en esta investigación confirman que existe variabilidad de la morfometría facial en relación a la forma del arco maxilar, en individuos de la ciudad de Corrientes. A través de los resultados se pudo analizar el biotipo facial y la forma de arco más frecuente dentro de la muestra, como también su frecuencia en sexo.

Estudios antropométricos citados por diferentes autores que tratan la identificación del biotipo facial datan de comienzos del siglo XIX, haciendo referencia en esa época, a las diferencias raciales que enfatizaron la superioridad de las poblaciones humanas europeas ^17^^-^^25^. En 1957, Martin y Saller ^12^ determinaron el biotipo facial como la anchura desde el cigomático derecho al cigomático izquierdo multiplicada por cien y dividida por la altura de la cara desde el punto nasión hasta el punto mentón. Del Sol ^2^ postula que hay factores que pueden producir variaciones morfológicas faciales, tales factores pueden ser socioculturales, genéticos y locales en lo que respecta a biotipo facial. Bedoya ^7^ por su parte sostiene que las características faciales y el desarrollo facial de los individuos siguen complejos patrones de desarrollo influenciado por varios factores, genético, medioambiental, sociocultural, generando de esta manera un patrón de crecimiento específico dentro de una población determinada. 

Este trabajo de investigación se realizó en individuos de la ciudad de Corrientes, utilizando el índice morfológico facial definido por Rakosi e Irmtrud, quienes consideran que a pesar de que puede influir en la determinación del biotipo facial el grupo étnico, este factor no estuvo presente. 

En la muestra estudiada se encontró que el biotipo facial preponderante es el mesoprosopo, seguido del leptoprosopo y euriprosopo, en coincidencia con los resultados encontrados por Solarte *et al*. ^20^. Similares resultados fueron hallados por Del Sol ^2^, con mayor prevalencia de mesoprosopo, seguida por euriprosopo y en menor proporción para el leptoprosopo. No se encontró el mismo comportamiento en el estudio de Núñez y Herrera ^6^ en donde el resultado obtenido fue mayor para el leptoprosopo, seguido por mesoprosopo y, por último, el euriprosopo.

La diversidad de resultados nos lleva a compartir lo estudiado por Bedoya *et al*. ^7^ que señalan que la forma facial está determinada por la herencia multifactorial, es decir, no solo por los genes, sino que también influyen factores ambientales y culturales generando de esta manera un patrón de crecimiento específico dentro de una población determinada.

En relación con el biotipo facial y la edad de los individuos, no se registró diferencia significativa en el grupo de estudio; en cambio, en relación con el sexo, se determinó que en los hombres el alto facial y ancho facial presentaron valores superiores a las medidas halladas en las mujeres. Por su parte, el índice morfológico facial no mostró diferencias en relación al sexo del paciente, en concordancia con el estudio realizado en individuos chilenos por Bustamante *et al*. ^21^.

Numerosos son los estudios que han mostrado diferentes métodos para determinar las formas de arco, que van desde la definición subjetiva, pasando por plantillas transparentes prefabricadas hasta el uso de funciones matemáticas y programas computarizados, todos encaminados a lograr el diagnóstico más exacto posible ^5^^,^^22^^-^^25^. La desventaja principal de las técnicas es su difícil aplicabilidad rutinaria en el consultorio. Un estudio realizado por Arai *et al*. ^26^ demostró que existe una correlación significativa entre la clasificación subjetiva de la forma de arco por parte del operador y la aplicación de ecuaciones polinómicas. En concordancia con este último trabajo, en esta investigación se aplicaron ambos métodos; por un lado, la ecuación de las medidas obtenidas de ancho anterior y posterior y, por el otro, la observación directa de los modelos. Los resultados obtenidos reflejan un predominio de la forma cuadrangular del arco superior, seguida de las formas ovoidal y triangular. 

Por otra parte, se logró determinar un índice para la longitud anterior del arco dental maxilar; el valor de este índice presentó concordancia con las formas del arco, definiendo un valor promedio entre 9 mm y 10,6 mm para la forma ovoide. Esto permitió inferir que valores inferiores están relacionados con la forma cuadrangular del arco, mientras que valores superiores con arcos triangulares. Estos resultados coinciden con lo observado por Solarte *et al*. ^20^, quienes hallaron esta relación en el maxilar inferior y no así en el superior.

Al relacionar la forma del arco superior con el sexo se encontró que en el sexo femenino predominó la forma cuadrangular, a diferencia del masculino, que presentó forma de arco ovoidal, seguida de la forma cuadrangular y, por último, la triangular, lo que contrasta con el estudio de Gutiérrez y Gutiérrez ^25^ donde la forma predominante del maxilar superior en el sexo femenino fue ovoide y en el sexo masculino cuadrangular, mientras que en otros estudios ^5^^,^^6^^,^^26^, no encontraron diferencias estadísticamente significativas entre sexos. 

El biotipo facial y la forma del arco dental son dos características físicas que se han estudiado y analizado en diferentes publicaciones. Se encontraron estudios comparativos sobre prevalencias y diferencias de forma de arco entre grupos poblacionales ^23^^,^^26^^-^^30^. En la muestra analizada, ninguno de los biotipos faciales presentó predominio de una forma de arco maxilar única.

Núñez y Herrera ^6^ en su estudio publican que los tipos faciales coincidieron con las formas de arco respectivas, mesoprosopo con ovoide, leptoprosopo con triangular y euriprosopo con cuadrada. Por el contrario, esta investigación en función de la forma del arco superior halló que la forma triangular se corresponde al biotipo facial euriprosopo, la forma ovoidal del arco al leptoprosopo y la forma cuadrangular se vinculó directamente con el mesoprosopo, aunque no se encontró estadísticamente una asociación significativa.

CONCLUSIONES

Los resultados obtenidos no permiten establecer un patrón antropométrico facial específico en los individuos de la ciudad de Corrientes. Asimismo, existe variabilidad morfométrica facial respecto a la forma del arco dentario superior.

## Fuente de financiamiento:

Secretaría General de Ciencia y Técnica de la Universidad Nacional del Nordeste (Argentina).
